# Next Generation Sequencing Mitochondrial DNA Analysis in Autism Spectrum Disorder

**DOI:** 10.1002/aur.1792

**Published:** 2017-04-17

**Authors:** Ashok Patowary, Ryan Nesbitt, Marilyn Archer, Raphael Bernier, Zoran Brkanac

**Affiliations:** ^1^ Department of Psychiatry and Behavioral Sciences University of Washington Seattle WA

**Keywords:** mitochondria, autism spectrum disorder, whole exome sequencing, single nucleotide variation, next generation sequencing

## Abstract

Autism is a complex genetic disorder where both de‐novo and inherited genetics factors play a role. Next generation sequencing approaches have been extensively used to identify rare variants associated with autism. To date, all such studies were focused on nuclear genome; thereby leaving the role of mitochondrial DNA (mtDNA) variation in autism unexplored. Recently, analytical tools have been developed to evaluate mtDNA in whole‐exome data. We have analyzed the mtDNA sequence derived from whole‐exome sequencing in 10 multiplex families. In one of the families we have identified two variants of interest in *MT‐ND5* gene that were previously determined to impair mitochondrial function. In addition in a second family we have identified two VOIs; mtDNA variant in *MT‐ATP6* and nuclear DNA variant in *NDUFS4*, where both VOIs are within mitochondrial Respiratory Chain Complex. Our findings provide further support for the role of mitochondria in ASD and confirm that whole‐exome sequencing allows for analysis of mtDNA, which sets a stage for further comprehensive genetic investigations of the role of mitochondria in autism. ***Autism Res*** *2017, 10: 1338–1343*. © 2017 International Society for Autism Research, Wiley Periodicals, Inc.

## Introduction

Mitochondria are known as the powerhouses of the cell as within the respiratory chain complex (RCC) they generate the majority of cellular energy through a series of oxidative phosphorylation reactions to form ATP [Saraste, 1999]. Any disruptions in RCC can cause perturbation in cellular energy production, potentially leading to a disease state [Smeitink, van den Heuvel, & DiMauro, 2001]. The genes coding for proteins involved in RCC are located in both, nuclear DNA (nDNA) and mitochondrial DNA (mtDNA) [Taylor & Turnbull, 2005]. The mitochondrial genome is a closed circular double stranded DNA molecule of ∼16,569 nucleotides that encodes 37 genes, 13 of which encode core structural RCC components, 22 are transfer and 2 are ribosomal RNAs [Anderson et al., 1982]. Unlike nDNA, the mtDNA is maternally inherited and is present in multiple copies per cell [Spelbrink, 2010].

MtDNA mutations play a well‐documented role in rare mitochondrial disorders (MD) and accumulation of mtDNA mutations has been implicated in aging and common diseases such as neurodegenerative diseases, cancer and metabolic disorders [Schon, DiMauro, & Hirano, 2012]. Mitochondrial dysfunction most often affects tissues and organs with high‐energy demands such as brain, muscle, liver, heart, and kidney. More than 300 mtDNA protein coding and regulatory region variants have been reported to be associated with disease phenotypes, and 34 mtDNA variants have strong evidence for causality in rare neurodevelopmental diseases (http://www.mitomap.org, 2016, [Lott et al., 2013; Schon et al., 2012]).

The role of mitochondrial dysfunction has been postulated in autism spectrum disorder (ASD) as well [Legido, Jethva, & Goldenthal, 2013]. The prevalence of MD in ASD is estimated at 5%, which is at least 200 times higher than the general population (Frye and Rossignol, 2011). ASD has a complex genetic architecture with contribution from common and rare variants [de la Torre‐Ubieta, Won, Stein, & Geschwind, 2016; Willsey & State, 2015]. Current studies do not support the role for common mitochondrial variants and haplotypes in ASD [Hadjixenofontos et al., 2013]. However, case reports and small studies have demonstrated the presence of mtDNA deletions, nucleotide substitutions, and copy number changes in subjects with ASD [Legido et al., 2013; Rossignol & Frye, 2012]. In addition, recent exome sequencing and mitochondrial analysis of simplex autism cases found evidence for elevated pathogenicity for mtDNA variants with low heteroplasmy [Wang, Picard, & Gu, 2016], indicating that further genetic studies of mitochondrial role in ASD are needed.

Next generation sequencing provides an excellent opportunity to fully characterize mtDNA and evaluate its contribution to ASD. One such approach is the evaluation of mtDNA through off‐capture sequence evaluation following conventional whole‐exome sequencing (WES). Off‐target mtDNA reads obtained from WES data can be readily identified, mapped and the variants can be called using existing tools. In our study we have characterized mtDNA in ten ASD families to identify variants of interest (VOI) that might contribute to the phenotype. Following the principles of Mendelian genetics and parallels with MD, we defined VOIs based on variant frequency and function. Furthermore, in families with mtDNA VOIs we have analyzed nDNA RCC genes to evaluate mtDNA–nDNA interactions. To the best of our knowledge, this is the first next generation sequencing based study aimed at investigating mtDNA in subjects with familial autism.

## Methods

We selected samples from the NIMH genetics repository (https://www.nimhgenetics.org/) Autism Distribution 5. All families in the repository have ASD cases diagnosed with gold standard autism diagnostic observation schedule [Lord et al., 2012] and autism diagnostic interview‐revised [Rutter et al., 2003]. Most of the 1428 families in Distribution 5 are sibships with two affected siblings. In order to increase the probability that mtDNA variants contribute to ASD, we obtained extended families that are connected through maternal lineage and sibships with four or more affected cases. Choice of extended families was based on maternal inheritance of mtDNA and we selected multi‐sib families as a part of an unpublished ASD exome sequencing project.

Exomes were captured using Nimblegen SeqCap EZ Human Exome Library v2.0 (Roche, Basel, Switzerland) following manufacturer's recommendation. Exome enrichment, sequencing and base calling were performed at the University of Washington Genome Sciences Center for Mendelian Genomics as described before [Chapman et al., 2015]. We used MToolbox to extract mitochondrial reads, call variants and identify mitochondrial haplogroups [Calabrese et al., 2014). Picardtool was used for filtering out the PCR duplicates and GATK IndelRealigner was used for read realignment around indels.

The hypothesis for our study was that in each family we can identify a small number of rare mtDNA VOIs that are shared between all affected cases within family. To identify VOIs we filtered out variants that define individual haplogroups and mtDNA variants with frequency ≥0.01 in 1000Genome data set (1000G) downloaded from MitoTool [Fan & Yao, 2011]. In addition to frequency we required VOIs to affect protein coding mtDNA (missense, frameshift, stop) and be present in all affected subjects in a family. The mtDNA VOI were confirmed with Sanger sequencing as described [Chapman et al., 2015]. Pathogenicity prediction and annotation for VOI were determined with MitImpact, a collection of pre‐computed pathogenicity predictions for all nucleotide changes that cause non‐synonymous substitutions in human mitochondrial protein coding genes [Castellana, Ronai, & Mazza, 2015].

To assess interactions with nDNA in families with mtDNA VOIs, we analyzed 83 nDNA RCC genes as defined by HGNC [Gray, Yates, Seal, Wright, & Bruford, 2015] (http://www.genenames.org/). For nDNA RCC genes SNVs were annotated with ANNOVAR as described before [Rubinstein et al., 2016], and VOIs defined using the same variant frequency and function criteria as for mtDNA.

## Results and Discussion

We sequenced four families with affected maternal cousins and six families with four or more affected siblings in NIMH Distribution 5 repository for a total of 35 individuals (25 male, 10 female) (Fig. S1). Mapping and assembly of reads onto the mitochondrial genome resulted in an average coverage of ≥98% of mtDNA bases with a mean read depth of 69× (Table 1). Overall we have identified 255 mtDNA variants, and after haplogroup and frequency filtering 72 rare variants remained. In each family, between 0 and 8 rare variants were shared for a total of 45 shared variants, 5 of which met the VOI criteria and were confirmed with capillary sequencing (Table 1). The nDNA analysis of four families with mtDNA VOI identified 10 shared variants including one nDNA VOI. List of nDNA RCC genes with shared variants is presented in Table S1. Five VOI present in 4 mtDNA genes, *MT‐ND5*, *MT‐CO1*, *MT‐CO3* and *MT‐ATP6*, and one nDNA VOI in *NDUFS4* are shown in Table 2. Exome sequences are deposited in NDAR (https://ndar.nih.gov/edit_collection.html?id=1919).

**Table 1:** Summary of Mitochondrial Genome Analysis from the Exome Sequences

Family ID	Haplogoup	No. of individuals	MT genome coverage	Avg read depth	Variants	Rare variants
152‐HSC0079	M10a1	3	94.98	46.47	18	4
72‐1397	A2w1	2	96.67	27.26	35	9
72‐1921	H1c	2	98.95	43.08	11	5
74‐0672	K1a	2	99.73	78.23	32	7
61‐2457	H3a	4	99.01	78.72	11	3
63‐302	H6a1b2	4	99.38	76.72	17	8
72‐1650	C1b9	5	99.23	101.98	41	10
74‐0327	H15a1	4	99.71	80.33	18	8
74‐0733	U4b1b1a	4	99.27	73.26	27	11
74‐0700	U5a1b	5	98.91	84.63	35	7

*Note*. Rows 1–4 are affected cousin families; rows 5–10 are multiple affected Sibs families.

**Table 2:** VOIs Identified in Our Study

Family ID	Genomic position	DbSNP ID	Ref/Alt	Gene	AA pos	AA sub	1000G frequency	SIFT Score	Polyphen2 Score	CADD Score
74‐0733	MT:13528	rs55882959	A/G	*MT‐ND5*	398	Tau/Asp	0.0019	0.66	0.12	9.242
74‐0733	MT:13565	rs56039545	C/T	*MT‐ND5*	410	Ser/Phe	0.0009	0.46	0.01	21.7
74‐0327	MT:6253	rs200165736	T/C	*MT‐CO1*	117	Met/Thr	0.0084	0.37	0	0.099
74‐0700	MT:9667	rs41482146	A/G	*MT‐CO3*	154	Asn/Ser	0.0084	0.57	0.14	2.017
72‐1397	MT:8896	rs202120082	G/A	*MT‐ATP6*	124	Ala/Thr	0.0028	0.39	0	11.55
72‐1397*	5:52899293	NA	C/G	*NDUFS4*	37	Thr/Ser	NA	0.1	0.01	18.9

*Note*. SIFT (Sorting Intolerant From Tolerant) score predicts impact of amino acid substitutions based on the degree of conservation in sequence alignments derived from closely related sequences. Scores <0.05 are considered deleterious. PolyPhen‐2 (Polymorphism Phenotyping v2) score predicts impact of a variant on the structure and function of a human protein using eight sequence‐based and three structure‐based predictive features. Scores >0.95 are considered probably damaging. CADD (Combined Annotation Dependent Depletion) score is a framework that integrates multiple annotations into one metric by contrasting variants that survived natural selection with simulated mutations. Higher CADD scores are considered more deleterious and variants with CADD score ≥ 10 are predicted to be the 10% most deleterious substitutions in the genome. None of the VOIs had all 3 bioinformatics predictions consistent with deleterious effects on phenotype.

*This variant was identified in the nuclear gene of the mitochondrial respiratory chain complex.

**Figure 1:**
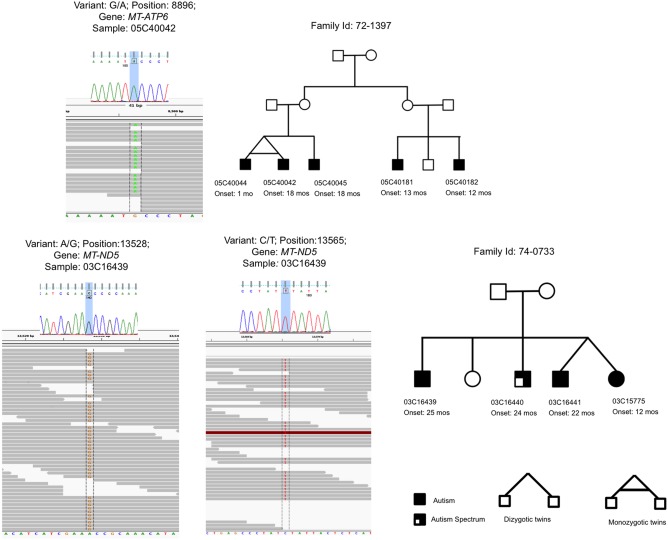
Capillary and next‐generation sequencing of mtDNA VOIs and family pedigrees. (**A**) c.8896G > A in the family 72‐1397. (**B**) c.13528A > G and c.13565C > T in the family 74‐0733. The variant capillary sequencing confirmation and next generation sequencing reads viewed with Integrative Genome Viewer for mtDNA variants are presented on the left; Family pedigrees are on the right.

The most compelling finding in our study was the identification of two *MT‐ND5* VOIs, c.13528A > G A13528G (Tau398Asp) and c.13565C > T (Ser410Phe), in multi‐sib family 74‐0733 (Fig. 1). The Tau398Asp variant was first reported in two unrelated patients that had Leber Hereditary Optic Neuropathy (LHON OMIM #53500) “like” ocular symptoms [Batandier, Picard, Tessier, & Lunardi, 2000], and later together with Ser410Phe mutation in one additional LHON [Petruzzella et al., 2012] and one Mitochondrial Myopathy, Encephalopathy, Lactic Acidosis, and Stroke‐like episodes (MELAS OMIM#540000) family [McKenzie et al., 2007]. LHON is a disease that usually manifests in adulthood and is restricted to eye, and MELAS is a severe multisystem disorder with childhood onset. In LHON family [Petruzzella et al., 2012], in addition to affected proband, Tau398Asp and Ser410Phe variants were present in unaffected mother and sister indicating incomplete penetrance. Both variants are associated with U4B1B haplogroup and have population frequency of 0.0019 and 0.0009 respectively in 1000G database. In subjects with both variants, extensive studies of mitochondrial function were performed [McKenzie et al., 2007; Petruzzella et al., 2012]. The fibroblasts with *MT‐ND5* A13528G and C13565T exhibited decreased mitochondrial membrane potential and increases lactate production. Furthermore, to exclude contribution of nDNA genes, the transmitochondrial cybrid fusion studies have demonstrated reduction of mitochondrial membrane potential and decrease in RCC linked respiration. Although for the two variants we have identified in *MT‐ND5* gene bioinformatics predictions of variant impact are not uniformly consistent with pathogenic role, the reported functional studies have demonstrated that the variants affect mitochondrial function and bioenergetics competence, which increases likelihood that variants have effects on phenotype.

Our second finding of interest was the identification of two RCC VOIs in a cousin family 72‐1397, rare mtDNA c.8896G > A (Ala126Thr) in *MT‐ATP6* (Fig. 1) and novel nDNA Thr37Sser *NDUFS4* variant. For the variants we identified in *MT‐ATP6* and *NDUFS4* bioinformatics prediction of functional impact are discordant as well (Table 2), and currently there are no published studies of in vitro variant effects. However, the identification of two VOIs in the same family brings the additional possibility that their combined effects can result in mitochondrial dysfunction [Anderson et al., 2011].

Available clinical data for four families with rare protein‐coding shared variants are provided in Supplementary Information. One of the limitations of our study is that we do not have extensive clinical information on affected subjects or genotype information for parents and unaffected relatives. Thus, we are not able to assess if affected subjects have clinical or laboratory characteristics of MD nor estimate variant penetrance. An additional limitation of our study is that our samples were derived from lymphoblastoid cell lines. Although no differences in mtDNA mutation profile between lymphoblastoid cell lines and whole blood DNA were reported [Diroma et al., 2014]; such a difference might be present. However, although brain tissues could be more informative for evaluating mtDNA in ASD, our focus on shared VOIs assumes that the variants we have identified are inherited and present in all tissues including brain tissues.

Our work further highlights difficulties in interpreting genetic findings and establishing genotype phenotype correlation for RCC variants. The MD are clinically and genetically heterogeneous and frequently caused by RCC mutations. MD manifestations are broad with age at onset ranging from the prenatal period to adulthood and severity ranging from prenatal lethality and severe multisystemic neurodevelopmental disorders such as MELAS to milder single organ phenotypes such as non‐syndromic hearing loss and LHON. The MD spectrum of phenotypes, such as developmental delay, loss of skills, seizures and abnormalities in musculoskeletal, endocrine and gastrointestinal systems overlaps with ASD [Frye & Rossignol, 2011]. This makes it plausible that in a subset of ASD subjects mitochondrial dysfunction is a pathogenic mechanism leading to the clinical phenotype. Further analyses of mtDNA and nDNA RCC and other genes involved in mitochondrial function in adequately powered samples are needed to evaluate the role of mitochondria in ASD. Such analysis should also take into account the combined effects of mitochondrial variation and gene interactions. The genes encoding mitochondrial proteins are excellent candidates for a “synergistic heterozygosity” genetic mechanism that postulates that combined effects in multiple steps of a pathway or process may lead to disease [Vockley, Rinaldo, Bennett, Matern, & Vladutiu, 2000]. Such a mechanism was experimentally demonstrated in mice with genes involved in mitochondrial fatty acid β‐oxidation [Schuler et al., 2005]. However, to strengthen the evidence linking variants that are thought to result from synergistic heterozygosity, functional data will be needed for large number of variants and their combinations. This necessitates high throughput functional assays [Gasperini, Starita, & Shendure, 2016]. In addition, detailed phenotypic assessment of ASD subjects with mitochondrial variants is critical to provide further evidence for mitochondrial contribution. Detailed phenotypic assessment of such ASD individuals should include comprehensive multisystem evaluation, biochemical testing and neuroimaging that are part of MD diagnostic process [Parikh et al., 2015]. Finding a higher prevalence of MD phenotypic signature in ASD subjects with mitochondrial variants would further strengthen the evidence for involvement of mitochondria in ASD. In addition, as specific therapeutic interventions for mitochondrial diseases are available and in development [Distelmaier, Haack, Wortmann, Mayr, & Prokisch, 2016], understanding of mitochondrial contributions to autism could rapidly translate to better treatments and outcomes for ASD patients with mitochondrial dysfunctions.

## Supporting information

Additional Supporting Information may be found in the online version of this article at the publisher's web‐site:

**Figure S1**. Pedigree of the Exome Sequenced Families. A: Cases related through their mothers. B: Affected siblings in nuclear family.
**Table S1**. Variants identified in the 83 nDNA mitochondrial respiratory complex chain genes and shared by the affected exome of family. 1000Genome frequencies are only for “European” population. **dbSNP132**: database of Short Genetic Variation version 132.
