# Supplementary material for: Next Generation Sequencing Mitochondrial DNA Analysis in Autism Spectrum Disorder
Source: Autism Res. 2017 Apr 17;10(8):1338–43. doi: 10.1002/aur.1792 (PMC5573912; doi:10.1002/aur.1792)
Supplement: Supplementary file 1 — Figure S1. Pedigree of the Exome Sequenced Families. A: Cases related through their mothers. B: Affected siblings in nuclear family. Table S1. Variants identified in the 83 nDNA mitochondrial respiratory complex chain genes and shared by the affected exome of family. 1000Genome frequencies are only for “European” population. dbSNP132: database of Short Genetic Variation version 132. [file AUR-10-1338-s001.docx]

**Clinical data for 4 exome Sequenced Families**

**Family 74-0733**

***03C16439***: 6 year old male with a diagnosis of ASD. He has a history of regression and no history of seizures.

***03C16440***: 3 year old male with a diagnosis of ASD. He has no history of regression and no history of seizures.

***03C16441***: 2 year old male member of a dizygotic twin pair with a diagnosis of ASD. He has a history of regression and no history of seizures.

***03C15775***: 2 year old female member of a dizygotic twin pair with a diagnosis of ASD. She has no history of regression and no history of seizures.

**Family 74-0327**

***03C17075***: 9 year old male with a diagnosis of ASD confirmed by ADI and ADOS. He has a history of regression and no history of seizures. He is verbally fluent and has a nonverbal IQ score of 75.

***03C17074***: 8 year old male with a diagnosis of ASD confirmed by ADI and ADOS. He has a history of regression and no history of seizures. He uses phrase speech to communicate and has a nonverbal IQ score of 50.

***03C17072***: 6 year old male with a diagnosis of ASD confirmed by ADI and ADOS. He has no history of regression and no history of seizures. He is verbally fluent and has a nonverbal IQ score of 107.

***03C17071***: 10 year old male with a diagnosis of ASD confirmed by ADI and ADOS. He has a history of regression and no history of seizures. He is verbally fluent and also has a nonverbal IQ score of 107.

**Family 72-1397**

***05C40044***: 7 year old male member of a monozygotic twin pair with a diagnosis of ASD confirmed by ADI and ADOS. He has no history of regression and no history of seizures. He is verbally fluent and has a nonverbal IQ score of 103.

***05C40042***: 7 year old male member of a monozygotic twin pair with a diagnosis of ASD confirmed by ADI and ADOS. He has no history of regression and no history of seizures. He is verbally fluent and has a nonverbal IQ score of 125.

***05C40045***: 3 year old male with a diagnosis of ASD confirmed by ADI and ADOS. He has no history of regression and no history of seizures. He is minimally verbal.

***05C40180***: 12 year old male with a diagnosis of ASD confirmed by ADI and ADOS. He has no history of regression and no history of seizures. He is verbally fluent and has a nonverbal IQ score of 97.

***05C40182***: 7 year old male with a diagnosis of ASD confirmed by ADI and ADOS. He has no history of regression and no history of seizures. He is verbally fluent and has a nonverbal IQ score of 119.

**Family 74-0700**

***03C17045***: 9 year old female with a diagnosis of ASD confirmed by ADI and ADOS. She has no history of regression and no history of seizures. She is verbally fluent and has a nonverbal IQ score of 110.

***03C17041***: 9 year old female with a diagnosis of ASD confirmed by ADI and ADOS. She has no history of regression and no history of seizures. She is verbally fluent and has a nonverbal IQ score of 114.

***03C17042***: 3 year old female with a diagnosis of ASD confirmed by ADI and ADOS. She has no history of regression and no history of seizures. She uses phrase speech and has a nonverbal IQ score of 119.

***03C17040***: 4 year old male with a diagnosis of ASD confirmed by ADI and ADOS. He has no history of regression and no history of seizures. He uses phrase speech and has a nonverbal IQ score of 110.

***03C23184***: 2 year old male with a diagnosis of ASD confirmed by ADI and ADOS. He has a history of regression and no history of seizures. He is minimally verbal.

Supplementary Figure1: Pedigree of the Exome Sequenced Families. A: Cases related through their mothers. B: Affected siblings in nuclear family.

A


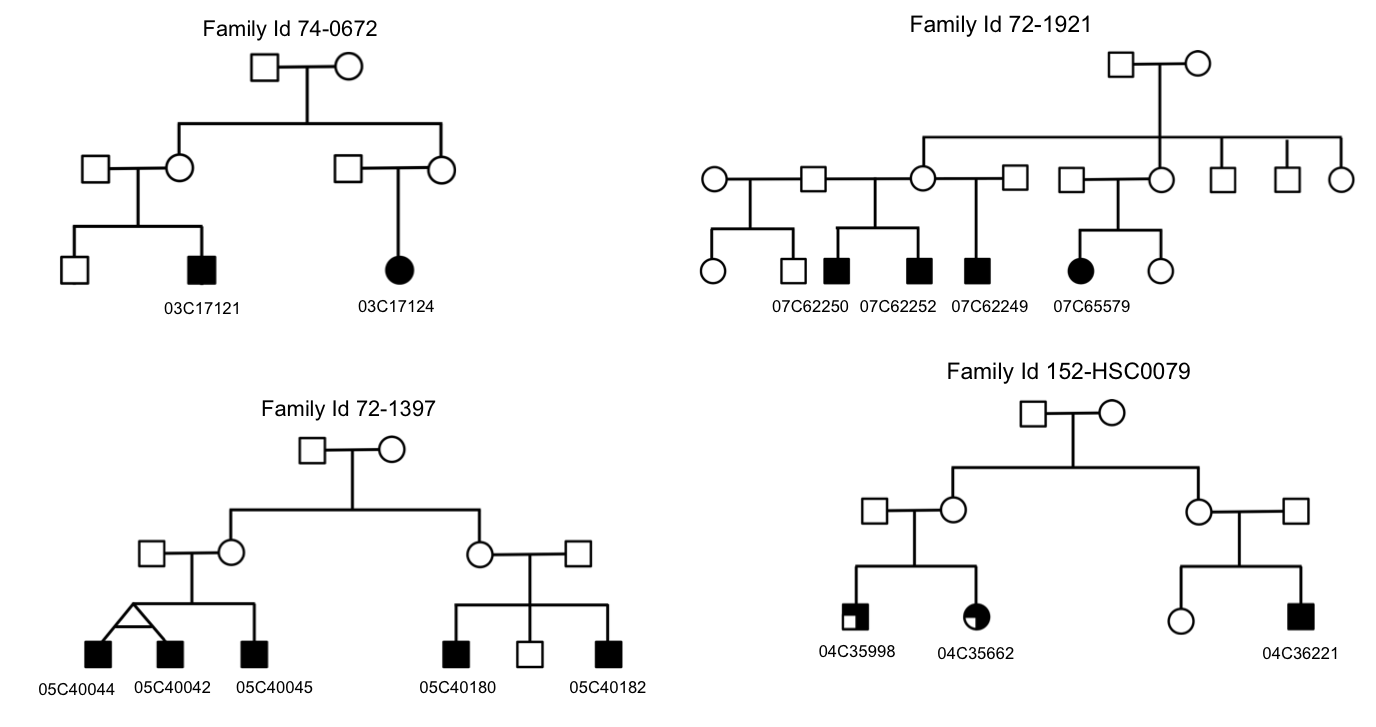


B


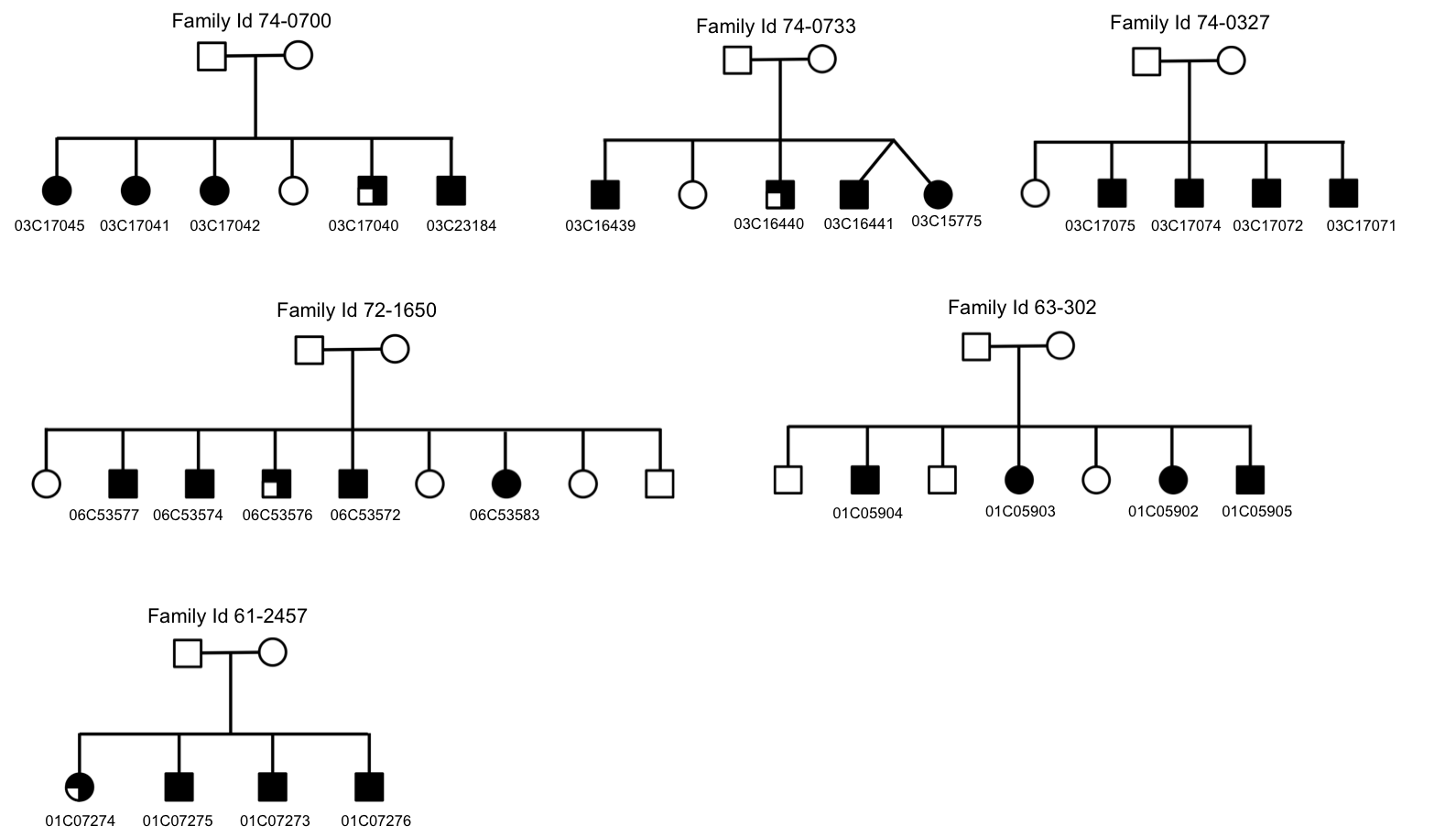


Supplementary Table 1: Variants identified in the 83 nDNA mitochondrial respiratory complex chain genes and shared by the affected exome of family. 1000Genome frequencies are only for “European” population. **dbSNP132**: database of Short Genetic Variation version 132.

| **Family Id** | **Position** | **Ref/ Alt** | **Gene** | **AA Change** | **1000Genome Frequency** | **dbSNP132** |
| --- | --- | --- | --- | --- | --- | --- |
| 74-0327 | chr5:256509 | G/A | SDHA | V609I | 0.11 | rs6962 |
| 74-0327 | chr8:125562029 | C/T | NDUFB9 | P90S | 0.09 | rs10195 |
| 74-0327 | chr18:9117867 | T/C | NDUFV2 | V29A | 0.82 | rs906807 |
| 74-0327 | chr19:1388538 | C/T | NDUFS7 | P23L | 0.61 | rs1142530 |
| 74-0327 | chr21:44324365 | G/A | NDUFV3 | D415N | 0.59 | rs10595 |
| 74-0700 | chr1:28564279 | A/G | ATPIF1 | R63G | 0.31 | rs9508 |
| 74-0700 | chr18:9117867 | T/C | NDUFV2 | V29A | 0.82 | rs906807 |
| 74-0700 | chr19:1388538 | C/T | NDUFS7 | P23L | 0.61 | rs1142530 |
| 74-0700 | chr19:29704010 | A/C | UQCRFS1 | S6A | 0.91 | rs8100724 |
| 74-0733 | chr1:28564279 | A/G | ATPIF1 | R63G | 0.31 | rs9508 |
| 74-0733 | chr18:9117867 | T/C | NDUFV2 | V29A | 0.82 | rs906807 |
| 74-0733 | chr19:1388538 | C/T | NDUFS7 | P23L | 0.61 | rs1142530 |
| 74-0733 | chr21:44324365 | G/A | NDUFV3 | D415N | 0.59 | rs10595 |
| 72-1397 | chr1:28564279 | A/G | ATPIF1 | R63G | 0.31 | rs9508 |
| 72-1397 | chr5:52899293 | C/G | NDUFS4 | T37S | NA | NA |
| 72-1397 | chr18:9117867 | T/C | NDUFV2 | V29A | 0.82 | rs906807 |
| 72-1397 | chr19:1388538 | C/T | NDUFS7 | P23L | 0.61 | rs1142530 |
| 72-1397 | chr21:44324329 | G/A | NDUFV3 | E403K | 0.02 | rs61746238 |
